# MRI geometric distortion: Impact on tangential whole‐breast IMRT

**DOI:** 10.1120/jacmp.v17i5.6242

**Published:** 2016-09-08

**Authors:** Amy Walker, Peter Metcalfe, Gary Liney, Vikneswary Batumalai, Kylie Dundas, Carri Glide‐Hurst, Geoff P Delaney, Miriam Boxer, Mei Ling Yap, Jason Dowling, David Rivest‐Henault, Elise Pogson, Lois Holloway

**Affiliations:** ^1^ Centre for Medical Radiation Physics, University of Wollongong Wollongong NSW Australia; ^2^ Liverpool and Macarthur Cancer Therapy Centres NSW Australia; ^3^ Ingham Institute for Applied Medical Research, Liverpool Hospital Sydney NSW Australia; ^4^ South Western Clinical School, University of New South Wales Sydney NSW Australia; ^5^ Institute of Medical Physics, School of Physics, University of Sydney Sydney NSW Australia; ^6^ Collaboration for Cancer Outcomes Research and Evaluation, Liverpool Hospital Liverpool NSW Australia; ^7^ School of Medicine, University of Western Sydney Sydney NSW Australia; ^8^ Department of Radiation Oncology Henry Ford Health System Detroit MI USA; ^9^ Commonwealth Scientific and Industrial Research Organisation Computational Informatics, Australian E‐Health Research Centre Brisbane Australia

**Keywords:** magnetic resonance imaging, geometric distortion, breast IMRT

## Abstract

The purpose of this study was to determine the impact of magnetic resonance imaging (MRI) geometric distortions when using MRI for target delineation and planning for whole‐breast, intensity‐modulated radiotherapy (IMRT). Residual system distortions and combined systematic and patient‐induced distortions are considered. This retrospective study investigated 18 patients who underwent whole‐breast external beam radiotherapy, where both CT and MRIs were acquired for treatment planning. Distortion phantoms were imaged on two MRI systems, dedicated to radiotherapy planning (a wide, closed‐bore 3T and an open‐bore 1T). Patient scans were acquired on the 3T system. To simulate MRI‐based planning, distortion maps representing residual system distortions were generated via deformable registration between phantom CT and MRIs. Patient CT images and structures were altered to match the residual system distortion measured by the phantoms on each scanner. The patient CTs were also registered to the corresponding patient MRI scans, to assess patient and residual system effects. Tangential IMRT plans were generated and optimized on each resulting CT dataset, then propagated to the original patient CT space. The resulting dose distributions were then evaluated with respect to the standard clinically acceptable DVH and visual assessment criteria. Maximum residual systematic distortion was measured to be 7.9 mm (95%<4.7mm) and 11.9 mm (95%<4.6mm) for the 3T and 1T scanners, respectively, which did not result in clinically unacceptable plans. Eight of the plans accounting for patient and systematic distortions were deemed clinically unacceptable when assessed on the original CT. For these plans, the mean difference in PTV $V_{95}$ (volume receiving 95% prescription dose) was 0.13±2.51% and −0.73±1.93% for right‐ and left‐sided patients, respectively. Residual system distortions alone had minimal impact on the dosimetry for the two scanners investigated. The combination of MRI systematic and patient‐related distortions can result in unacceptable dosimetry for whole‐breast IMRT, a potential issue when considering MRI‐only radiotherapy treatment planning.

PACS number(s): 87.61.‐c, 87.57.cp, 87.57.nj, 87.55.D‐

## I. INTRODUCTION

Magnetic resonance imaging (MRI) is being used more frequently in radiotherapy planning due to superior soft‐tissue contrast.^1^, ^2^ While MRI remains investigational at the present time in breast planning, it potentially offers greater soft‐tissue delineation than CT scans.^(3)^ With the growing interest in the utilization of MRI for radiotherapy treatment planning, the geometric distortions associated with MRI require consideration.^(4)^ Distortions due to nonlinearities in the gradient coils and inhomogeneities in the $B_{0}$ field increase in magnitude with increasing distance from the MRI isocenter.^(5)^ The patient introduces additional effects, such as chemical shift and susceptibility due to the variations in magnetic properties of different tissues. Although the implementation of vendor‐supplied distortion correction algorithms can reduce the system‐related distortions, they are not completely removed.^(6)^ Several methods can be utilized to correct for patient‐specific distortions, including acquiring two images with reversed gradient direction^(7)^ and $B_{0}$ mapping;^(8)^ however, clinical implementation of these can be challenging and is not routinely implemented.^(4)^ Acquiring images with a high imaging bandwidth (BW) can be used to minimize the distortions due to chemical shift and magnetic susceptibility. However, an increased BW results in a number of trade‐offs including a reduced signal‐to‐noise ratio (SNR), which could be problematic in certain signal‐challenging sites and acquisition techniques.^(9)^

Investigations into the dosimetric impact of MRI geometric distortions on the radiotherapy treatment planning (RTP) process have been conducted for brain and prostate patients. Brain studies focus on a small field of view (FOV) at the center of the scanner where distortions are minimal.^10^, ^11^ The prostate studies were conducted on low‐magnetic field‐strength scanners,^12^, ^13^, ^14^ or on phantom geometry.^(15)^ Prott et al.^(16)^ suggested that these effects may have more influence on breast cancer treatment, where the anatomy extends out into regions of higher geometrical inaccuracies.

The aim of this study was to investigate the effect of MRI geometric distortions on the dose distribution in tangential whole‐breast, intensity‐modulated radiation therapy (IMRT). The impact of residual system distortions alone was compared for two different scanners with different field strengths and configurations. The influence of the combined distortion arising from the system and the patient was also investigated on the higher field‐strength system.

## II. MATERIALS AND METHODS

### A. Phantom data

#### A.1 Image acquisition

Residual system distortion maps were obtained from two separate MRI scanners with different configurations and field strengths using specific phantoms.

An in‐house developed 3D phantom comprising of vitamin E capsules within a plastic housing structure^(5)^ was scanned on a closed, wide‐bore 3T Siemens Magnetom Skyra (Siemens Medical Systems, Erlangen, Germany). A 2D spin echo sequence (voxel size $1.56\times 1.56\times 2{\text{mm}}^{3},500\times 500\times 252{\text{mm}}^{3}$ FOV, 445 Hz/pixel, TE/TR/flip angle 12 ms/2760 ms/90°) was acquired with vendor 2D distortion correction algorithm applied (to match acquired patient images — detailed below). The air‐phantom interface had minimal impact on the distortion assessment in the region of interest for this study since the interface occurs at the FOV edges and all patient anatomy is encompassed within this volume. A corresponding CT of this phantom was acquired to provide the undistorted geometry of the phantom.

Using a 1.0 T Panorama high‐field open vertical‐bore system (Philips Medical Systems, Best, The Netherlands), a vendor‐supplied 3D distortion phantom consisting of docusate sodium capsules within a foam structure^(17)^ was scanned using a 3D T1 spoiled gradient echo sequence (voxel size $0.938\times 0.938\times 2{\text{mm}}^{3},450\times 450\times 400{\text{mm}}^{3}$ FOV, 385 Hz/pixel, TE/TR/flip angle: 3.77 ms/30 ms/60°, 3D correction algorithm applied). ACT of this phantom was also acquired. Standard shimming was performed over the whole volume prior to each image acquisition on both scanners.

#### A.2 Quantification of residual system distortions

To quantify systematic distortions, the CT and MR phantom images from each scanner were registered. A robust rigid registration method^(18)^ was applied to compensate for changes in position between modalities, then a deformable multiscale (four levels) B‐spline registration algorithm^(19)^ was used to account for nonlinear MRI deformations. To promote smoothness, the spacing of the final B‐spline control point grid was adjusted by selecting the largest value that would allow accurate alignment of the phantom markers postregistration, as determined by visual assessment. We identified 25 mm and 10 mm as the best control point spacing parameters for registering MRI‐CT images for the 3T and the 1T phantom images, respectively. The registration results were manually validated by verifying that the phantom markers were accurately aligned to within one voxel.^(5)^

### B. Patient data

#### B.1 Image acquisition

Datasets for 18 patients who underwent whole‐breast, external‐beam radiotherapy (12 right‐sided, 6 left‐sided) were obtained. The images were acquired for a breast MRI‐CT contouring study and were retrospectively obtained for the purposes of this investigation. Patients were positioned on both CT and MRI using the same treatment simulation setup on a flat table and in a head‐first supine position. Patients were set up on an MRI‐compatible wing board (MTWB09 Wingboard, CIVCO Medical Solutions, Orange City, IA) with no angular incline for both imaging modalities. The patient position on the wing board was recorded per clinical practice for ensuring consistency between simulation and treatment. Noncontrast CT images were acquired on a Philips Brilliance Big Bore scanner (Philips Health Care, Cleveland, OH) (voxel size $0.98\times 0.98\times 2{\text{mm}}^{3},500\times 500\times 252{\text{mm}}^{2}$ FOV). Field length was determined on individual patient requirements. The MRI was acquired on the same day as CT, on the aforementioned 3T MRI scanner only for comparison with the acquired phantom images. T2‐weighted 2D turbo spin echo images were acquired (voxel size $0.6\times 0.6\times 2{\text{mm}}^{3},400\times 400\;{\text{mm}}^{2}$ to $450\times 450\;{\text{mm}}^{2}$ FOV, TE/TR/flip angle 83–88 ms/2480–7910 ms/150°–167°), with standard tune‐up $B_{0}$ shimming localized to the imaging volume, per the study imaging protocol. Patients were requested to shallow‐breathe during the image acquisition. The sequence acquired ran for 6 min/14 s. No ghosting was observed in these images, indicating minor effects of breathing motion. A standard 18‐channel surface coil was placed on top of foam coil bridges custom‐designed within the department to ensure the coils did not displace the anatomy. Images were prospectively corrected using the vendor‐supplied 2D gradient distortion correction algorithm (3D not available for the sequence utilized at commencement of study). The initial 10 patients were acquired with a pixel BW of 230 Hz/pixel to provide good SNR. The final eight patients were acquired with a pixel BW of 450 Hz/pixel, reducing patient distortions but at the expense of SNR.

Clinical target volume (CTV) and seroma volumes for each patient based on the CT images were contoured by an experienced radiation oncologist. Lung, heart, contralateral breast, and external patient volumes were contoured by an experienced senior planner.

#### B.2 Quantification of residual systematic and patient‐induced distortions

Distortion maps representing the combined residual systematic and patient‐induced distortions were estimated by registering the patient CT images with the corresponding patient MRI (3T only). The deformable B‐spline registration process used for the phantom images was also used for the patient data, but with a final control point grid spacing of 15 mm, which was found to give adequate accuracy at a realistic smoothness level over the entire FOV. Each registered image was visually assessed for accuracy, with particular focus around the breast and chest wall regions where anatomical landmarks (ribs, outer breast surface) were accurate to within 1 voxel. To validate the deformable registration further, a 3D volume of the breast volume (incorporating the skin surface and the chest wall) was automatically segmented by thresholding in ITK‐SNAP (version 3.4.0) for each dataset. The Dice similarity coefficient (DSC) was then calculated in MILXView for the volumes generated on the original planning CT and MRI, then compared with the DSC for the deformed CT and MRI. The DSC has a value of 1 for perfect agreement and 0 when no overlap is present.

### C. Image distortion

**Figure 1:**
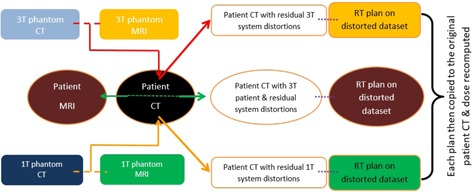
Methodology schematic showing the image distortion processes for the phantom and patient datasets.

To investigate the impact of MRI geometric distortion, each patient CT (and corresponding contours) was distorted in three ways, using the phantom‐computed distortion maps (3T and 1T) and using the combined residual system and patient‐induced distortion maps (3T only) (Fig. 1). The phantom data allowed investigations on the potential dosimetric variations between MRI scanners of different configurations in whole‐breast RTP without the confounding factor of patient‐induced effects. With the 3T, using both phantom‐ and patient‐based distortion maps allowed for a comparison between the system‐related effects and combined system and patient‐related effects.

In all cases, contour variation was assessed for the CTV, seroma, contralateral breast, combined lung, and heart volumes. The DSC was obtained between original and distorted contours to investigate their overlap using:

(1) $$\begin{array}{rl} & DSC=2\times [\text{intersection volume}/(\text{original contour volume}+\\ & \;\text{distorted contour volume})] \end{array}$$

### D. Treatment planning

After registration, four different CT datasets were considered (Fig.1):

1. The original CT,
2. A CT dataset deformed according to the 3T residual system distortions,
3. A CT dataset deformed according to the 1T residual system distortions, and
4. A CT dataset nonrigidly registered to the patient geometry on the 3T.

Our study utilized a scripted tangential IMRT technique reflecting the current clinical practice of predominately tangential beams and the emerging trends of IMRT utilisation.^20^, ^21^ For each of the simulated CT datasets, inverse‐planned hybrid IMRT plans were created using an automated script in Pinnacle Version 9.6 (Philips Radiation Oncology Systems, Milpitas, CA) to a prescription of 50 Gy in 25 fractions in accordance with standard clinical protocols. The script was designed to establish gantry angle and determine the field size and beam modulation, with limited degrees of freedom ($\text{maximum number of segments}=8,\text{minimum segment area}=9{\text{cm}}^{2},\text{minimum monitor units}=6$, minimum leaf pairs 6, and minimum leaf end separation=3cm). Beam angles with an isocenter located at the center of PTV (defined as CTV expansion of 1 cm superior–inferior, 0.5 cm left–right, and 0.5 cm anterior–posterior) were automatically determined based on the location of the lungs, PTV, and contralateral breast contours for each patient dataset.^(22)^ Manual beam‐angle adjustment was permitted (within 4°) if the automatically generated angle restricted obtaining acceptable DVH criteria.

After completion of the script, each plan was individually optimized by adjusting the appropriate IMRT optimization parameters to achieve clinically acceptable DVH criteria^(23)^ (Table 1) and visual acceptance by an experienced senior planner. No other optimization parameters were changed, to avoid variations in planning technique. Visual acceptance was based on department clinical practice and ensured a balanced distribution of any hotspots in the plans, an acceptable assessment of the 100% and 95% isodose curves compared with the DVH criteria, and ensured the dose was not compromised near skin surface. Qualitative visual assessment was conducted to confirm homogeneity of the dose distribution within the PTV, as well as ensuring high dose levels did not occur outside the PTV and that the maximum dose occurred within the PTV. The aim of the script was to automate the planning process and reduce the subjectivity of planning. Any additional changes required to optimize the plan were performed by the same planner for consistency among all datasets. After final optimization, multileaf collimator (MLC) banks were manually adjusted on appropriate beam segments at the anterior flash section to allow 2 cm overshoot anteriorly. This resulted in a hybrid IMRT technique with (on average) 80% of the dose delivered through open beams with a 2 cm anterior overshoot and 20% of the dose delivered through IMRT beams.

**Table 1:** The DVH criteria for plan evaluation (based on RTOG guidelines and clinical experience).

*Target/OAR Structure*	*Ideal Criteria*	*Acceptable Criteria*
PTV	>95%receives>47.5Gy	>95%receives>45Gy
	Maximal 1cc≤53.5Gy	Maximal 1cc≤55Gy
Seroma	>99%receives>47.5Gy	>99%receives>46Gy
	Maximal1cc≤55Gy	Maximal1cc<57.5Gy
Combined Lung	<10% receives 10 Gy	<10% receives 10 Gy
	Mean dose<10Gy	Mean dose<10Gy
Ipsilateral Lung	<15%receives>20Gy	<20%receives>20Gy
Heart	Mean<4Gy	Mean<5Gy
Contralateral Breast	Maximal1cc<1.86Gy	Maximal1cc<3.10Gy

### E. Dosimetric evaluation

Once plans were optimized on each distorted dataset, the beams from these plans were exported and then imported onto the original CT image with the beam isocenter placed at the center of the undistorted PTV, simulating a treatment setup position. Tangential beams were recomputed using fixed monitor units (MU), beam weights, and dose grid resolution. No additional optimization was performed at this stage, in order to determine the impact of the distortion. The dose criteria (Table 1) were then tabulated. Each of these plans was then assessed as a pass or fail based on DVH and visual acceptance criteria. Plans meeting the DVH and visual assessment criteria were generated on all 18 original (undistorted) CT images. This provided a baseline from which the distortion impact could be tested.

## III. RESULTS

### A. Residual system distortion

The maximum residual system distortions were 7.88 mm (95% below 4.67 mm) over a $500\times 500\times 254{\text{mm}}^{3}$ FOV on the 3T and 11.87 mm (95% below 4.56 mm) over a $475\times 380\times 420{\text{mm}}^{3}$ FOV on the 1T. The largest distortions were observed at the FOV edges. The target registration error between the capsule centers was within one voxel.

### B. Contour deformation

**Figure 2:**
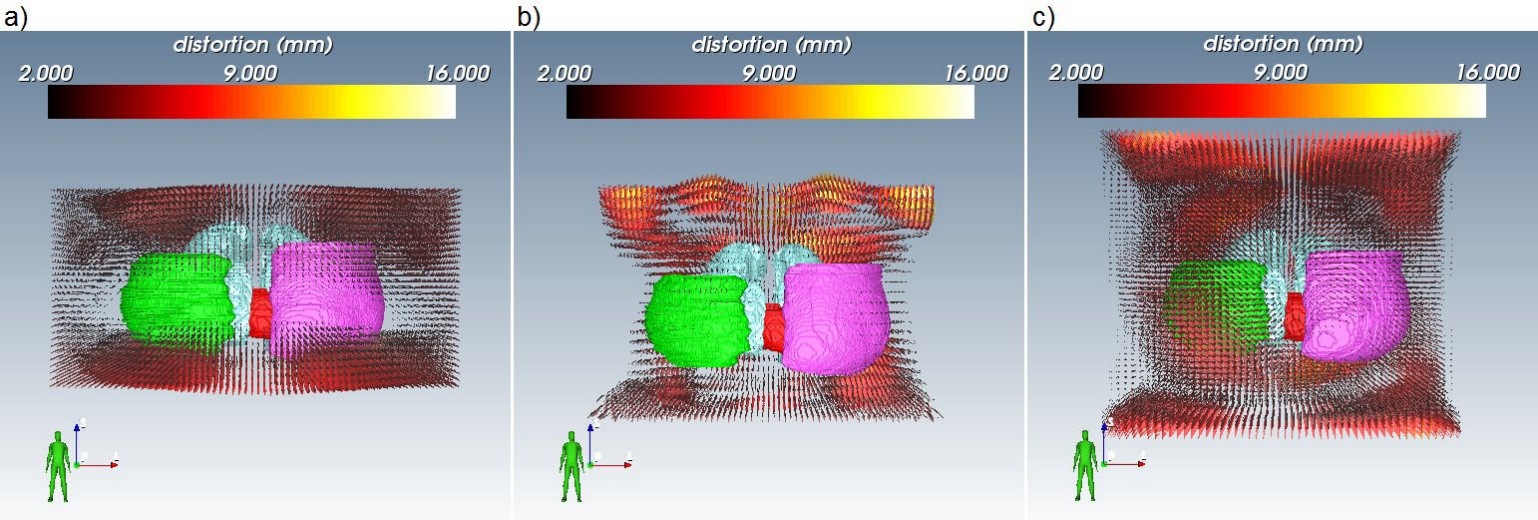
Coronal comparison of the CTV (green), contralateral breast (pink), lung (blue) and heart (red) volumes relative to distortions ≥2mm for (a) the 3T phantom, (b) an example 3T patient dataset (incorporating patient and system related distortions as well as set‐up uncertainties), and (c) the 1T phantom. Scales set relative to maximum distortion in (b). Visualization by http://smili‐project.sourceforge.net/.

The average size of contoured volumes is displayed in Table 2. The maximum distortion observed in each contour and the DSC overlap between distorted and undistorted contours is presented for each distortion type, averaged across all patients. Distortion vector fields displaying where distortions ≥2mm are shown in Fig. 2 for all three deformations, relative to the contour locations. Maximum distortions for the patient measurements (inclusive of setup errors) (Fig. 2(b)) were larger than the residual system distortions alone (Fig. 2(a)).

**Table 2:** Variations in the contour volumes and overlap comparisons between original and distorted contour volumes. Phantom data consists of residual system distortions, while patient scans also include patient‐specific distortions and setup uncertainties.

*Scanner*	*Contour*		*Mean Undistorted CTV* Volume±2σ (cm^3^)	*Mean Maximum* Distortion±2σ *(mm) Within Contour*	*Mean* DSC±2σfor *Each Contour*
3T phantom	CTV	(R)	796.0±737.7	3.0±1.2	0.97±0.01
		(L)	485.8±641.3	2.6±0.8	0.97±0.01
	Seroma	(R)	28.0±87.2	1.0±1.1	0.96±0.08
		(L)	11.0±16.0	1.2±0.4	0.91±0.12
	CB	(R)	974.9±774.6	3.7±1.5	0.97±0.01
		(L)	585.0±515.5	2.9±0.6	0.97±0.01
	Combined Lung		2462±1220	3.0±1.3	0.98±0.00
	Heart		477.6±134.4	1.4±0.6	0.99±0.01
1T phantom	CTV	(R)		2.9±0.5	0.97±0.01
		(L)		2.5±1.4	0.95±0.02
	Seroma	(R)		2.3±0.5	0.96±0.10
		(L)		2.0±0.4	0.90±0.07
	CB	(R)		3.4±1.2	0.96±0.01
	(L)			2.8±0.5	0.96±0.02
	Combined Lung			4.9±1.7	0.97±0.01
	Heart			2.0±0.2	0.98±0.01
As above					
3T patient	CTV	(R)		5.9±4.6	0.94±0.02
		(L)		6.6±6.9	0.91±0.06
	Seroma	(R)		3.7±4.0	0.82±0.19
		(L)		4.6±4.2	0.74±0.16
	CB	(R)		6.4±3.1	0.94±0.02
		(L)		7.0±4.4	0.91±0.06
	Combined Lung			11.3±8.6	0.93±0.04
	Heart			9.1±7.1	0.92±0.05

CB=Contralateral Breast; R=Right‐sided patient; L=Left‐sided patient; σ=standard deviation; DSC=Dice similarity coefficient.

### C. Deformable registration validation

The mean DSC between the breast volume on the original planning CT and the patient MRI was 0.938 (range 0.909 – 0.964). Following registration, the mean DSC between the deformed planning CT and the MRI increased to 0.962 (range 0.912 – 0.980) indicating that the deformed CT images were well matched to the MRI.

### D. Dosimetric impact

**Figure 3:**
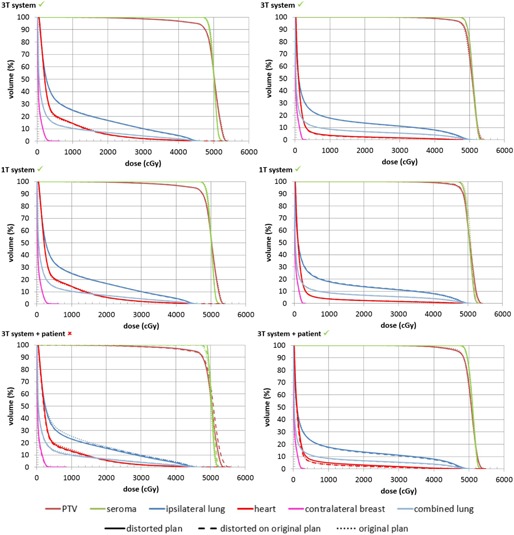
DVHs for two patients (left and right) showing the variation in dose coverage variation that occurs when incorporating different distortion factors. ✓=distorted plan met/did not meet criteria when recomputed on original image.

Mean variations in DVH criteria between distorted plans optimized on the distorted dataset compared to that of the distorted plan recomputed on the original datasets are shown in Table 3. All plans optimized on either the 3T and 1T phantom distorted datasets passed both the DVH and visual assessment when recomputed on the original CT dataset. Of the 18 plans optimized on the CT datasets that were deformably registered to the 3T patient images, four right‐sided and four left‐sided patients failed the clinical tolerances when recomputed on the original dataset (two failed DVH, four failed visual, two failed DVH and visual assessments). Figure 3 compares DVHs for two patients for the combined 3T distortions (one pass, one fail), 3T systematic, and 1T systematic distortions. Of the eight patients that were rendered clinically unacceptable based on our dosimetric acceptance criteria, five were imaged at the lower pixel bandwidth and three were imaged at the higher bandwidth.

**Table 3:** Quantitative assessment of the DVH parameters and the clinical plan acceptance. All numbers were computed based on dose optimized on distorted datasets less dose when recomputed on undistorted dataset.

*Dose Criteria*		Mean Difference±2σ(3T phantom)	Mean Difference±2σ(1T phantom)	*Mean Difference* ±2σ(3T patient)
*Right*				
PTV	$V_{47.5\text{Gy}}$	0.2±1.2%	0.5±1.8%	0.3±2.5%
	Max 1cc	−0.2±0.3 Gy	0.0±0.4 Gy	0.2±0.7 Gy
Seroma	$V_{47.5}\;\text{Gy}$	0.1±0.8%	−0.1±0.5%	0.2±1.7%
	Max 1cc	0.1±0.2 Gy	0.2±0.4 Gy	0.1±0.8 Gy
Ipsilateral Lung	$V_{20}\;\text{Gy}$	−0.1±0.4%	−0.5±0.9%	0.0±0.3 Gy
Heart	Mean	−0.0±0.1 Gy	0.8±2.7%	−0.0±0.3Gy
CB	Max 1 cc	−0.0±0.1Gy	−0.0±0.3Gy	0.1±0.6Gy
*Left*				
PTV	$V_{475\text{Gy}}$	−0.8±1.6%	−0.3±1.4	−0.7±1.9%
	Max 1cc	−0.0±0.1Gy	0.0±0.2Gy	0.5±1.0Gy
Seroma	$V_{47.5\text{Gy}}$	−0.5±1.6%	−0.5±2.0%	−0.4±1.7%
	Max 1cc	−0.2±0.6Gy	−0.0±0.2Gy	0.2±0.9Gy
Ipsilateral Lung	$V_{20\text{Gy}}$	−0.2±0.5%	−0.3±0.6	−0.3±1.8%
Heart	Mean	0.1±1.3Gy	−0.1±0.2Gy	−0.4±0.9Gy
CB	Max 1 cc	−0.0±0.1Gy	−0.1±0.2Gy	0.3±2.4Gy
Combined Lung	$V_{20\text{Gy}}$	−0.1±0.2%	−0.1±0.4%	−0.1±0.7%
Plan pass/fail	Overall	18✓/0×	18✓/0×	10✓/8×

CB=Contralateral Breast; ✓=plan met acceptable criteria; ×=plan did not meet acceptable criteria.

## IV. DISCUSSION

This work investigated the impact of system‐ and patient‐related distortions on MRI‐based whole‐breast IMRT planning. Results have shown that residual system distortions can vary greatly between two different scanners, both of which are utilized for RTP simulation. However, the effect of these distortions alone was found to have minimal impact on photon dosimetry. Residual system distortions, combined with patient‐related distortions (including chemical shift and susceptibility), potentially have a larger impact on dosimetric variation, requiring consideration if utilizing patient geometry as observed on MRI in the radiotherapy planning process.

Most modern scanners come with the option to apply geometric correction algorithms to account for the gradient nonlinearities in 2D and/or 3D. Though distortions may not be completely removed, a reduction in systematic distortions is shown when applying the 2D correction over no correction,^(6)^ and when applying the 3D correction over the 2D correction.^(5)^ Patient MRIs were acquired with a vendor‐supplied 2D correction algorithm as the 3D correction was unavailable at commencement of patient imaging. However, these images acquired with the 2D algorithm provide a “worst case” scenario and the dosimetric impact of the 3D correction can be expected to be smaller.

Both the wide, closed‐bore 3T and the vertical, open‐bore 1T performed similarly in terms of dosimetric impact in terms of the residual system distortion effects as measured by phantoms. Despite the known increase in gradient nonlinearity distortions with increased distance from isocenter, residual distortions of laterally positioned breasts did not adversely impact dosimetry. Maximum distortion values observed within contoured regions for the 3T patient‐distorted datasets were on average 3.8 times greater than those corresponding to the phantom data alone, indicating that the patient‐induced distortions (and associated setup errors) were larger than those of the residual system distortions.

Datasets incorporating both patient‐ and residual system‐related distortions were found to have greater dosimetric impact than the residual system distortions alone, and therefore these will need to be considered if utilizing MRI in the whole‐breast radiotherapy planning process. On average, plans generated on patient‐distorted images were more inhomogeneous with increased hot and cold regions when recomputed on the original CT geometry. The probability of a distorted plan passing or failing did not correlate with laterality, extent of contour distortion, patient size, or pixel bandwidth. Placing a patient within the magnetic field leads to geometric distortions arising from chemical shift and susceptibility artefacts. These effects are the result of variations in the magnetic properties of tissues within the body. This is an issue for breast imaging where there are interfaces between glandular tissue, fatty tissue, muscle, ribs, lungs, patient surface, and air. The chemical shift due to difference in water and fat signals is 440 Hz at 3T (220 Hz at 1.5T), resulting in field perturbations of 3.5 parts per million (ppm). This corresponds to a geometric shift of 2 pixels/4 mm in the higher bandwidth scans (up to 4 pixels/8 mm in the lower bandwidth scans) for this study. Field perturbations due to susceptibility between different tissues can be as large as 6 ppm.^(4)^ Increasing the BW further could have benefit in reducing the susceptibility effects, though this would come at the expense of the SNR, which may limit clinical utility. These preliminary results show that, even in high BW scans, some plans failed to meet the dosimetric criteria.

These findings vary from investigations on the prostate and brain, where system and patient distortions were not found to have a significant impact on dosimetry.^12^, ^13^, ^14^ However, these studies were conducted on scanners of lower field strengths (with concomitant reduction in patient effects^(24)^) and the anatomical regions of interest are located in the center of the FOV where system effects are negligible. Additionally, these sites generally have smaller air‐tissue interface regions near the target. In contrast, our current study can be expected to produce a greater problem owing to the peripheral location of breast tissue and higher field strength.

While vendor‐supplied distortion corrections are essential to minimize the dosimetric impact of system‐related distortions, our results suggest that patient‐specific correction methods may be helpful in reducing the dosimetric impact of geometric distortions on the planning process. Techniques such as $B_{0}$ mapping^(8)^ or the acquisition of two images of reversed frequency‐encode direction^(9)^ that could reduce patient susceptibility effects were not part of the acquisition protocol since this data was acquired retrospectively. This will be investigated in future work.

Due to the retrospective dataset utilized for this study, separating the errors due to the patient distortion from those due to setup variations is challenging. As such, a number of techniques were utilized to minimize the impact of setup errors on this study, as is performed clinically within the department for patient treatments. MRI and CT images were acquired on the same day to reduce variations due to changing patient anatomy over time. All patient setup and imaging was performed and recorded by an experienced radiation therapist to ensure consistent setup between both imaging modalities and treatment. The patient CT and MRIs were rigidly registered prior to deformable registration to reduce setup errors to within the tolerance values to minimize dosimetric variations. Systematic and random setup errors for whole‐breast radiotherapy have been reported as 1–2 mm and 2–3 mm, respectively.^(25)^ Harron et al.^(26)^ reported that translational shifts of 5 mm would result in a less than 5% variation in the target volume receiving 95% and 107% of the prescribed dose. A study by George et al.^(27)^ investigated the dosimetric impact on breast radiotherapy for no, shallow, normal, and heavy breathing (0, 0.5, 1, and 2 mm displacement, respectively). They found that, when compared to no breathing motion, the PTV D_95_ was reduced by 5%, 8%, and 10% for shallow, normal, and heavy breathing, respectively. DVH variations across all three datasets within our study were less than these reported setup values. The hybrid IMRT technique utilized for this study (80% dose through open beams, 20% dose through IMRT beams) has also been found to the most be robust treatment technique when dealing with setup errors for whole‐breast radiotherapy.^25^, ^28^

The image registration presents another potential study limitation since the plans are being generated on manipulated images and structures. The registration accuracy was within a voxel‐size within the chest region of interest. When registering the patient CT and MRIs, there were some differences around the patients' arms due to slight positional variations. Since these were well out of the dose calculation region, these variations were not considered. Image registration is also a point of consideration when utilizing both CT and MRIs in the planning process.^(29)^

Distortion mitigation must be addressed if moving towards MRI‐only planning where a patient CT would not be available for reference. Although our results showed the influence of the scanners alone had minimal impact, it is important to quantify the geometric accuracy of each system. This is especially important for future MRI‐linac treatment units where magnet and gradient specifications may be worse, as well as on higher field‐strength systems where the patient effects will be more problematic.

The results presented are specific to whole‐breast IMRT treatments. While breast treatment volumes extend into FOV regions where geometric distortions are largest, the dosimetric impact from residual system distortions alone appear small. Since breast is a relatively homogeneous tissue structure, the photon interactions may be more forgiving of these geometric variations. In anatomical sites with multiple structures, the dosimetric impact of residual distortions may be larger due to more complex planning methods and tissue variations. These factors may be more important when considering partial‐breast radiotherapy and will be considered in the future. In a partial‐breast treatment, the dosimetric impact may be increased with smaller treatment volumes and more targeted fields.

## V. CONCLUSIONS

Combined MRI‐systematic and patient‐related distortions may result in unacceptable dosimetric variations for whole‐breast IMRT if considering MRI‐only radiotherapy treatment planning. Residual system distortions alone had minimal impact on the dosimetry variations for two different scanners of differing configurations and field strengths. Improved methods to account for patient‐specific distortion would be beneficial to minimize dosimetric variations if using MRI alone in the treatment planning process.

## ACKNOWLEDGMENTS

The authors would like to thank Robba Rai and Ewa Juresic for their knowledge and assistance with the scanning of the phantom on the 3T; Haijie Jin's work on the breast planning script; Shekar Chandra for software help for image visualization; Dean Cutajar for setup assistance for computer systems and software; and the department of radiation oncology, Henry Ford Health System, for scanning and supplying phantom data for this study. The authors would like to acknowledge funding assistance from Cancer Australia and The National Breast Cancer Foundation project grant number 1033237, Liverpool and Macarthur Cancer Therapy Centres trust fund scholarship (AW), 1 R01 CA204189‐01A1 (CGH) and the NSW Cancer Institute Leaders Program (PM).

## COPYRIGHT

This work is licensed under a Creative Commons Attribution 3.0 Unported License.
